# Role of Arbuscular Mycorrhizal Fungi in Regulating Growth, Enhancing Productivity, and Potentially Influencing Ecosystems under Abiotic and Biotic Stresses

**DOI:** 10.3390/plants12173102

**Published:** 2023-08-29

**Authors:** Abdul Wahab, Murad Muhammad, Asma Munir, Gholamreza Abdi, Wajid Zaman, Asma Ayaz, Chandni Khizar, Sneha Priya Pappula Reddy

**Affiliations:** 1 Shanghai Center for Plant Stress Biology, CAS Center for Excellence in Molecular Plant Sciences, Chinese Academy of Sciences, Shanghai 200032, China; 2 University of Chinese Academy of Sciences, Beijing 100049, China; muradbotany1@uop.edu.pk; 3 State Key Laboratory of Desert and Oasis Ecology, Xinjiang Institute of Ecology and Geography, Chinese Academy of Sciences, Urumqi 830011, China; 4 Department of Chemistry, Government College Women University, Faisalabad 38000, Pakistan; asmach994@gmail.com; 5 Department of Biotechnology, Persian Gulf Research Institute, Persian Gulf University, Bushehr 75169, Iran; abdi@pgu.ac.ir; 6 Department of Life Sciences, Yeungnam University, Gyeongsan 38541, Gyeongbuk, Republic of Korea; 7 Faculty of Sports Science, Ningbo University, Ningbo 315211, China; asmaayaz@bs.qau.edu.pk; 8 Institute of Molecular Biology and Biochemistry, University of the Lahore, Lahore 51000, Pakistan; chandnikhizar@gmail.com; 9 The UWA Institute of Agriculture, University of Western Australia, Perth, WA 6009, Australia; sneha.pappulareddy@research.uwa.edu.au

**Keywords:** arbuscular mycorrhiza fungi, crop productivity, ecosystem, sustainable, soil

## Abstract

Arbuscular mycorrhizal fungi (AMF) form symbiotic relationships with the roots of nearly all land-dwelling plants, increasing growth and productivity, especially during abiotic stress. AMF improves plant development by improving nutrient acquisition, such as phosphorus, water, and mineral uptake. AMF improves plant tolerance and resilience to abiotic stressors such as drought, salt, and heavy metal toxicity. These benefits come from the arbuscular mycorrhizal interface, which lets fungal and plant partners exchange nutrients, signalling molecules, and protective chemical compounds. Plants’ antioxidant defence systems, osmotic adjustment, and hormone regulation are also affected by AMF infestation. These responses promote plant performance, photosynthetic efficiency, and biomass production in abiotic stress conditions. As a result of its positive effects on soil structure, nutrient cycling, and carbon sequestration, AMF contributes to the maintenance of resilient ecosystems. The effects of AMFs on plant growth and ecological stability are species- and environment-specific. AMF’s growth-regulating, productivity-enhancing role in abiotic stress alleviation under abiotic stress is reviewed. More research is needed to understand the molecular mechanisms that drive AMF-plant interactions and their responses to abiotic stresses. AMF triggers plants’ morphological, physiological, and molecular responses to abiotic stress. Water and nutrient acquisition, plant development, and abiotic stress tolerance are improved by arbuscular mycorrhizal symbiosis. In plants, AMF colonization modulates antioxidant defense mechanisms, osmotic adjustment, and hormonal regulation. These responses promote plant performance, photosynthetic efficiency, and biomass production in abiotic stress circumstances. AMF-mediated effects are also enhanced by essential oils (EOs), superoxide dismutase (SOD), peroxidase (POD), ascorbate peroxidase (APX), hydrogen peroxide (H_2_O_2_), malondialdehyde (MDA), and phosphorus (P). Understanding how AMF increases plant adaptation and reduces abiotic stress will help sustain agriculture, ecosystem management, and climate change mitigation. Arbuscular mycorrhizal fungi (AMF) have gained prominence in agriculture due to their multifaceted roles in promoting plant health and productivity. This review delves into how AMF influences plant growth and nutrient absorption, especially under challenging environmental conditions. We further explore the extent to which AMF bolsters plant resilience and growth during stress.

## 1. Introduction

Industrialization, urbanization, and globalization are shrinking the arable lands and declining agricultural production, leading to increased food demand for the rapidly growing population. In addition, changing climatic conditions have resulted in extreme weather conditions that ultimately lead to more droughts, high temperatures, and floods, affecting the food supply from agricultural systems [1]. There are several reasons, including that export crops often have a higher economic value. In contrast, biofuel crops offer a substitute for conventional fossil fuels as a source of energy. Furthermore, these crops are often more resilient to changing climates, making them a viable option in some areas [2]. These consequences have had far-reaching implications for our planet, with the effects of climate change becoming more pronounced with each passing day [3]. Sustainable agriculture, or agricultural systems that bind the use of natural resources to produce food in a way that minimizes the adverse effects of the production process on the environment is crucial to achieving this balance [4]. It is becoming increasingly clear that soil is an integral part of food production and a critical resource that must be managed carefully to ensure long-term economic and environmental sustainability [5,6]. A comprehensive overview of what we currently know about AMF and how it may be used to improve crop yields under both optimal and stressed conditions will be provided as part of our study. As part of our discussion, AMF was discussed as a way to improve crop growth.

By secreting more enzymes, bacteria facilitate nitrogen uptake by plants. However, there is an issue since oxygen destroys nitrogen-free enzymes. To combat this issue, bacteria interact with plants to develop a nodule root structure [7,8,9]. Many studies demonstrated the beneficial effects of AMF on crop yields, which will be discussed in this review with an in-depth analysis. These symbiotic relationships allow plants to obtain nutrients from the unavailable soil. In addition to boosting plant growth and well-being, fungi absorb phosphorus and other essential minerals from the soil. Due to the release of enzymes by the fungus, the plant can gain access to nutrients that would otherwise be inaccessible to it.

Moreover, the fungal part releases several hormones that aid the plant in developing an extensive root system. In exchange, the plant provides the fungus with sugar, the main component for energy and survival [10]. Agroecosystems are designed to minimize the number of external inputs, such as pesticides, chemical fertilizers, and water, while maximizing natural resources, such as sunlight and soil nutrients. This makes them more sustainable than traditional farming methods [11]. For instance, beneficial bacteria can fix nitrogen from the atmosphere, make it available to plants, and suppress soil-borne diseases [12]. They establish a mutualistic relationship with plant roots, providing essential minerals and water from the soil in exchange for photo-synthetically fixed carbon from the plant [13].

**Figure 1:**
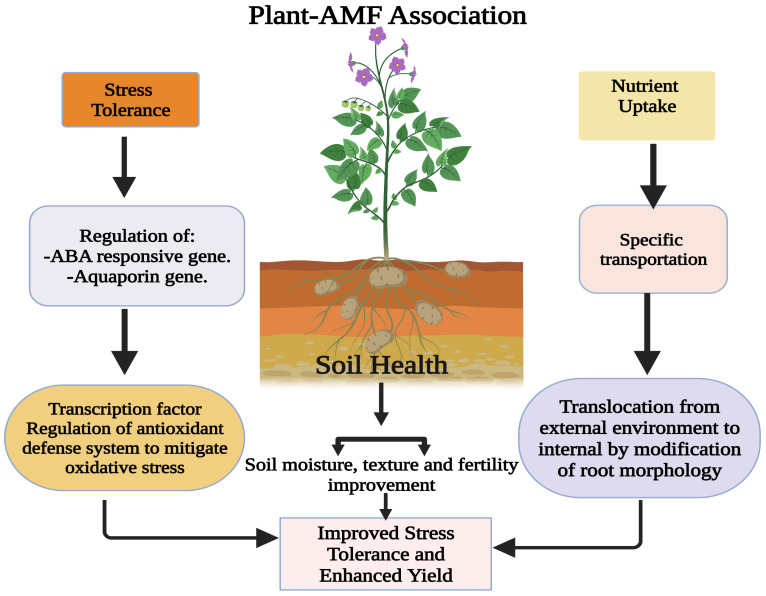
Beneficial microorganisms like Arbuscular mycorrhizal fungi enhance soil fertility and the ability of plants to uptake more nutrients.

Furthermore, these fungi are also known to provide critical benefits to the plants they associate with, such as improved water and nutrient uptake, increased resistance against diseases [10], and nutrient mobilization from organic substrates [14]; these interactions can lead to dramatic changes in the composition, structure, and functioning of plant communities, and a comprehensive understanding of these processes is essential for successful ecosystem conservation and management [15,16,17,18]. The imbalance of beneficial microorganisms caused by the disruption in the microbial population has led to soil deterioration and decreased crop production. Alterations in the composition of microbial communities can also result in changes in soil nutrient cycling and a reduction in soil water retention, leading to further soil degradation [19]. Microbes are essential for sustainable soil fertility management in nutrient cycling, pest management, and soil structure. This study aims to draw attention to the significant role that endomycorrhiza symbiosis can play as a provider of ecosystem services to ensure crop yield and assist in developing sustainable agriculture systems. Consequently, the general goal of this study is to review the work of AM growths on agricultural yield efficiency and biological system administration. Hence, understanding microbial interactions and how they interact with plants is critical to developing sustainable management of soil fertility and crop production (Figure 1) [20].

In return, the microbes receive a continuous supply of nutrients, allowing them to flourish and provide a healthier environment for the proper growth of plants [21]; for instance, plant roots often provide nitrogen and other essential minerals to their associated fungi, while the fungi provide the plants with the essential nutrients and water [22]. Moreover, AMF can also help plants resist environmental stress, such as drought and soil salinity, resulting in higher levels of plant growth and an increased rate of survival; by making the most of these technologies, smallholder farmers can not only maximize the yields of their crops but also enhance the sustainability of their farming practices for future generations [23] (Table 1).

Arbuscules, internal fungal structures in the root cortical cells, allow arbuscular mycorrhizal (AM) fungi to form strong relationships with a host plant [50]. According to current estimates, AM fungus started cooperating with host plants between 400 and 480 million years ago, facilitating the first terrestrial plant colonization of the land [51]. Approximately eighty per cent of terrestrial plant species are in relationships of close symbiosis with AM fungus [52] for various factors that benefit plants, such as nutrient acquisition, crop mass, yield increases, and reduced stress from abiotic pressures. AMF is a crucial and helpful gathering of soil accumulation that may significantly increase crop efficiency and ecological continuity in the production methods of new plants [53]. *Endomycorrhiza fuofs* allows the start of a mutualism relationship along with the root structure of eighty per cent of plant families; it just does not better the development of plants via enhanced absorption of phosphorus (P) available in the soil and other on-labile mineral nutrients necessary for the development of the plant; it also has ‘unhealthy’ effects on maintaining the collected soil, intended to stop erosion, and overcomes stress in plants due to abiotic and biotic factors [54,55]. AM fungi’s positive effects on plant execution and soil well-being are fundamental for agricultural ecosystems to be managed sustainably [56].

### 1.1. Economic Importance of Soil Microbes

However, as the “first green revolution,” beneficial soil microbes, generally, and AM in particular, have received significantly less attention [57,58,59,60]. Even though some offerings stand outside the marketplace and are hard to measure, the lowest approximate equivalent or outstrip worldwide gross countrywide outcomes [61]; a price tag of USD 190 billion has been calculated based on the value of goods and services provided by nature, including clean water, fertile soils, and pollination. This is double the worth of the gross national items of the world. According to present research, two critical environmental offerings, ‘formation of the soil’ and ‘cycling of nutrients, were expected to highlight USD 17.1 and 2.3 trillion US dollars. Simultaneously, most nations consume their charge frameworks to save the climate by limiting the number of exercises involved in the pollution they allow (such as the carbon tax) or to motivate the growth of rules that are in favour of the environment (Ecological Tax Reform); Costa Rica is the first nation to made a countrywide attempt to safe atmosphere offerings [62]. In 1996, this nation followed the rule (Forestry Law No. 7575), spotting four vital services supplied through the public woods: carbon elimination, hydrological offerings, biodiversity security, and attractive charm [63]. The rule set up a structure for the fee for atmosphere offerings, as outlined in a program authorized by the pagos por service Ambien (PSA) and managed through the fund provided by the National Forestry (FONAFIFO), which includes landlords and all other succeeding customers of the earth who agreed to charge environment offerings for twenty years and offer opposition through replanting, continuous management, conservation, and strategies of rebirth [64]. The availability of farming goods and environmental services is essential to human existence and the standard of life.

Nevertheless, new farming techniques that have significantly enhanced the world’s food contribution have unintentionally negatively affected the environment and resources [65,66]. In the context of advancement, new and beneficial procedures are needed to operate the Earth’s surrounding services and counter the lack of effort to hold necessary outcomes for renewable food manufacturing in the face of the enhancing populace worldwide [67]. Horticulture is the most significant connection between people and the climate; accommodating yield creation and natural respectability, reasonable harvest, and crop production is difficult for agribusiness and predetermination for landowners [68]. This demonstrates extending yield executives methodologies that enhance soil fertility, organic assortment, and crop production through developing agroecosystems that favour standard ecological methods and support efficiency in the long term [69].

Ecological administrations sustain soil attributes and plant well-being; soil flexibility in this environment is incredibly relevant [70]. Specifically, soil microorganisms that structure cooperative interactions with the roots of plants have attracted growing attention in rural examination and improvement since they provide a natural substitute to stimulate plant development and lower contributions to feasible editing arrangements [71]. The omnipresence of arbuscular mycorrhizal growths at the point of interaction among soil and plant roots makes them an essential and valuable association of soil biota. Their wholesome and non-dietary attributes significantly affect surrounding methods promoting farming yield creation and agroecological environmental administrations [72,73]. The proper management of environmental services provided by AM will positively influence the utilization and conservation of natural resources for the benefit of human societies [74]. Biomolecules have a backbone made of carbon, which is a necessary component of living organisms.

Nevertheless, too much CO_2_ in the atmosphere is thought to be dangerous. Thus, it is involved among the primary greenhouse gases [75]. Carbon sequestration captures carbon dioxide to lower atmospheric carbon dioxide levels [76]. The amount of carbon dioxide in the atmosphere directly impacts global climate changes, seriously threatening the entire biosphere [77]. The amount of CO_2_ has steadily risen in the atmosphere, with an average of approximately 385 ppm [78]. Some research teams have been performing evaluations to determine the most effective carbon sequestration technologies for a few decades. Two primary geological and biological carbon sequestration processes have been documented [79,80]. One of the critical processes of biological carbon sequestration is the assimilation of atmospheric CO_2_ through the biological activity known as photosynthesis. To reduce atmospheric CO_2_ levels, microbe-mediated CO_2_ uptake in the soil and plants is a crucial problem. The increase in photosynthetic rate is caused by bacterial populations in the leaf endosphere, phyllo sphere, and rhizosphere, despite photosynthesis being a natural process that consumes atmospheric CO_2_ [81]. Similar to how mammals develop symbiotic connections with microorganisms and have probiotics, plants have a variety of microbial communities and do the same [82].

### 1.2. Molecular Mechanisms of Plant-Microbe Interactions

Microbial communities linked with plants impact the operation of intricate bio-networks, such as complete food chains in many ecosystems [83]. Entophytic and endophytic interactions between plants and microorganisms have generally been discovered. However, these interactions are context-dependent, bidirectional, and complicated [84]. Despite some understanding of the relationship between plants and bacteria, scientific research has restricted attempts to use microbes as bio-stimulants or biofertilizers. Plant-associated microorganisms’ different molecular pathways to increase photosynthetic rate are being investigated [85]. By supplying specific bioactive chemicals, endophytes colonized in the interior tissues of plant parts help improve plant growth, yield, and disease resistance [86]. In contrast, the host plant provides carbon substrates to the microorganisms. Interestingly, a certain microbial strain prefers to colonize the rhizosphere when atmospheric CO_2_ levels are rising over those of other strains [87]. Improving carbon absorption by plants that rely on particular microbial strains would also help lower atmospheric CO_2_. For instance, the bacterial strain *Pseudomonas simiae* colonizes the rhizosphere much more quickly than the strain *P. putida* when the CO_2_ concentration is high [88]. In addition, well-documented microbe-mediation increases in photosynthetic rate in the tissues of plant leaves. The rhizosphere’s microbial population is solely reliant on plant signals. In addition to their fundamental tasks, plants’ roots are involved in various additional systems. Organic macromolecules called “root exudates” are discharged into the rhizosphere by roots, making up 5 to 40% of the plant’s photosynthetically absorbed carbon [89]. For instance, the root exudates contain substances like citric acid, fumaric acid, flavonoids, tryptophan, etc., that function as signalling molecules and draw advantageous microorganisms to the rhizosphere [90]. Under high CO_2_ conditions, plant roots release root exudates that attract beneficial microbes to colonize in the rhizosphere or within the plant tissues [88]. Similarly, when plants’ carbon sinks, such as respiration, growth, and storage, require them to store photosynthetically fixed carbon, they enlist the help of beneficial bacteria to improve the rate at which they fix carbon through photosynthesis [87]. When bacteria colonize the root system, root exudates play a role in the selection processes that favor certain bacteria over others. The photosynthetic pigments chlorophyll and carotenoids are the most abundant in the plant’s aerial portions, and it has been suggested that bacteria work as bio-stimulants to increase this concentration. By increasing the chlorophyll content, plant growth-promoting rhizobacteria (PGPR), Enterobacter sp., considerably accelerates the development of the Okra plant overall [91]. Similar advantages can be attained by intentionally introducing fungal endophytes into the plants. For instance, adding fungal endophytes to Coleus forskolin plants increased their yield by increasing their total photosynthetic rate [92]. Microbes increase chlorophyll content to increase the photosynthetic rate even under stress circumstances caused by metal elements. Lycopersicon esculentum, a plant growing under cadmium (Cd) stress, has a dramatically higher total chlorophyll content after being inoculated with *P. aeruginosa* or Burkholderia gladioli, according to Khanna et al.’s (2019) analysis. Microbes connected with plants can increase the expression of genes involved in photosynthesis and boost the effectiveness of the enzymes needed for biological carbon fixation [93]. In the plant Sedum alfredii, growing under Cd stress, Wu et al. (2018) showed that bacterial inoculants might enhance the effectiveness of photosynthetic enzymes including Rubisco, Ca^2+^-ATPases, and Mg^2+^-ATPase and upregulate the expression of photosystem related genes (SaPsbS and SaLhcb2) [94]. One of the primary purposes of stomata is carbon fixation, which enables the plant to absorb CO_2_ and exhale O_2_. There have been claims that bacteria can control stomatal conductance in plant leaves. Interestingly, some research discovered enhanced stomatal conductivity [95], while others found that it was reduced when the plants were colonized by certain microbes [96]. Carbon is removed from the environment and deposited in the soil due to increased CO_2_ sequestration from the air, improved photosynthesis, and augmented soil organic matter [97]. By boosting plant root development, microbial inoculants improve photosynthesis and deposit soil organic matter in the rhizosphere. Plant growth hormones are produced by root-associated microorganisms [98], enhance the expression of genes involved in root growth and development [92,99], and increase the concentration of photosynthetic pigments, chlorophyll, and carotenoids [100], all of which contribute to considerable organic matter deposition in the soil in the form of increased root yield. Reactive oxygen species (ROS) are produced in leaf tissues when photosynthetic pigments are overexcited or when plants are exposed to environmental challenges [101]. The released ROS reduces the photosynthesis rate by inhibiting photosynthetic enzyme activity [102]. Interestingly, *Rhizobiaceae* bacteria, some endophytes, *Trichoderma* sp., *Piriformospora indica*, arbuscular mycorrhizal fungi, and other beneficial microbes boost the expression of genes that make proteins that detoxify reactive oxygen species, as shown in Table 2 [103], thereby protecting the plants’ photosynthesis efficiency from ROS. Overall, plant development and physiology are significantly impacted by the CO_2_ uptake in plants mediated by microbes. For instance, it has been demonstrated that higher CO_2_ concentrations promote plant development by enhancing photosynthetic C input, nutrient and water utilization efficiency, and biomass product [104,105]. C fixation is therefore utilized in modern agriculture, especially in focused agricultural systems, as a “fertilizer” to increase plant yields. In order to increase plant productivity and lessen the effectiveness of the greenhouse gas, CO_2_, released by human activities, we must better understand the molecular mechanisms of plant–microbe interactions that enable plants to increase the efficiency of their photosynthetic processes as well as the role of various microbe-related factors in CO_2_ fixation.

## 2. Arbuscular Mycorrhizal Fungi Background

It is believed that many of the fungi in the Chromista group, such as slime molds and oomycetes, have the same ability to survive in extreme environments as fungi. This means that they are likely to be present in areas not usually explored by humans. Thus, their study is essential for understanding how it works in different environments and how diseases can be spread. Furthermore, since many fungi are parasites or symbiotes, previewing the literature can help us better understand the complex relationships between animals and fungi. It is also important to note that fungi do not possess animals’ digestive, nervous, or circulatory systems [160,161,162].

Furthermore, fungi reproduce by releasing spores or other reproductive cells, whereas animals reproduce sexually. In typical fungi, filamentous cytoplasms are surrounded by plasma membranes and cell walls called hyphae, similar to insect exoskeletons. Chitin is a flexible polysaccharide found in the fungi’s cell walls. In hyphae, several auxiliary cell walls are called “cross walls” or “septa” and are typically perforated by large pores, allowing ribosomes, mitochondria, and nuclei to pass through [163]. Mycelium has a multicellular structure that forms hyphae when it matures; it digests organic food externally before absorption by secreting enzymes that help it. The fungal mycelium can spread over large areas, allowing the plant root system to obtain phosphate and other minerals far away from nutrient-depleted walls known as “zones”. In contrast, the plant provides sugars to the fungus [164].

Among the many benefits of endomycorrhizal systems, plants can obtain more water and nutrients and increase protection against pathogens and droughts [165]. The mycelium breaks down organic matter in the soil and transfers nutrients to the plant, whereas the plant provides the mycelium with carbohydrates for energy; this relationship between the mycelium and the root tissue is mutually beneficial [166]. In addition to increasing plant nutrient absorption, AM fungus can make plants more resilient to abiotic stressors [167]. According to Spatafora et al. (2016), most AMF species are members of the Mucoromycota phylum’s Glomeromycotina subphylum [168]. This subphylum has 25 taxa and many orders, including the Glomerales, Archaeosporales, Paraglomerales, and Diversisporales [169]. To complete their life cycle, they consume lipids [18,170] and products from photosynthetic plants [171]. AMF shields plants against fungal diseases and enhances water and mineral nutrient intake from surrounding soils [172]. As a result, AMFs are essential for the ecosystem and plant production. It is impossible to overestimate the significance of these crops for sustainable agricultural development [173].

### 2.1. Sustainability of Agriculture and AM Fungi

By harnessing the natural abilities of the soil, it is possible to reduce the need for chemical inputs, which are costly and harmful to the environment. Additionally, reducing input overheads such as water and fuel can help reduce costs and increase efficiency [174]. Finally, preventing ecological contamination helps to protect the ecosystems in which agribusiness operates and can help to ensure sustainability in the end. An effective management system must be implemented and monitored to ensure the soil environment is suitable for crop growth while promoting beneficial soil microbes. This includes maintaining adequate moisture and nutrient levels and controlling nutrient leaching and compaction [175].

Other edaphic factors must be accounted for, such as the presence of nematodes and the application of organic or inorganic fertilizers. Because plants and microbes work together interestingly, mycorrhizal fungi significantly ensure sustainable agriculture [176]. A link between these symbiotic fungi in sustainable farming systems appears imperative, especially when certain ingredients are in short supply. AM is invaluable in mobilizing nutrients into usable forms under these conditions [177]. The extra-radical mycelium within this culture can be invaluable because AM fungal proliferation has enhanced soil quality and structural stability [178]. This has been shown to encourage plant growth. AM fungal proliferation is becoming an increasingly significant part of sustainable agricultural practices as it contributes to soil quality and structural stability. Utilizing these mycorrhizal associations would efficiently boost productivity by reducing chemical inputs such as insecticides and fertilizers [179]. The use of inorganic fertilizers on soils with low fertility has been spread widely, organic matter has been added, and practices such as fallow tilling and incorporation of leguminous crops have all been employed to improve soil ecosystems, boost soil microbial growth, and increase nutrient reuse to minimize external inputs while maximizing the effectiveness of those inputs [180].

This is why earthworms and microsymbionts are so helpful for managing soil ecosystems—they act as a form of natural fertilizer, helping to promote nutrient cycling in the soil and cultivating beneficial microbial associations [181]. Additionally, research has indicated that soil fungi have a variety of functions, such as aiding in decomposition and providing essential nutrients for plants. However, more research is needed to understand the full extent of their role in the soil ecosystem. It should be noted that mycorrhizae play a significant role in ecology [182]. It is because of their widespread distribution and potential contribution to nutrient cycling and biomass production by soil microbes. As a result, a crop’s germplasm must be tailored specifically to the environment where it will be grown [183]. In this way, the plant will receive the exact number of essential elements and minor nutrients needed for optimal growth if it grows in an optimal environment. This also allows the plant to cope with biotic and abiotic stresses like heat and drought, which can negatively affect crop yields. Because of this study, the symbiosis between plants and AMF existed long before plants colonized the land [184,185]. The fossil record indicates that there have probably been AMF on the land for at least 480 million years. This indicates that they likely influenced their success before plants were introduced to the land. As a result, they likely had a significant impact on terrestrial ecosystems during that period. This would suggest that they played a significant role in shaping them during that period [1,35,52,55,60,186,187].

### 2.2. AM Fungi in Crops Defense Mechanisms

AMF strengthens the plant’s immune system, giving it a more extraordinary ability to resist pests and pathogens more effectively [188]. In addition to providing nutrition to the plant, fungi also improve its growth and development, making it more likely for the plant to grow and develop more rapidly and effectively. Furthermore, fungi also produce compounds that act as antibiotics, which helps to protect the plant from specific pathogens [189]. This suggests that the colonization of plants by these beneficial microbes may be due to their ability to modify the plant’s defense pathways, allowing the microbes to colonize the plant without triggering an immune response. Additionally, the microbes may be able to access nutrients inaccessible to the plant or produce compounds that make the plant more resilient to environmental stress [103]. If mycorrhizal fungi colonize plants, a systemic priming effect can result from reassigning defense molecules/signals to the plants. This leads to a significantly higher crop defense DNA production when the plants are not colonized [190]. As well as improving plant nutrient uptake and growth, mycorrhizal colonization can also increase the attractiveness and quantity of plants and improve plant nutrient uptake and growth. Herbivores are also encouraged to consume plants that are abundant in nutrients, as this increases their availability of nutrients [191]. It has been demonstrated that crops colonized by AMF are more resistant to phloem-feeding insects than crops without AMF. Any farming management strategy involving AMF should include the notion that AMF symbionts may assist plants in mounting unspecific defenses against phloem-feeding herbicides. These mycelial networks can communicate between plants infested with aphids and plants not infested with aphids [192,193]. Thus, plants produce a rapid flow of aphid-repellent volatile compounds before they are attacked, thus preventing the spread of the disease and reducing productivity. Essentially, the mycorrhizal fungi form a symbiotic relationship with the roots of a crop, exchanging nutrients, water, and carbon for the crop’s nutrients. In addition, the fungi produce numerous secondary metabolites that are antibacterial and antifungal, which can assist in protecting the plant from microorganism-related diseases. The fungi can strengthen the plant’s ability to resist certain diseases and stimulate the plant’s immune system [191].

When pathogens are present in the root zone, this resistance mechanism can be triggered, causing the plant to release phenolic compounds that inhibit these pathogens’ growth. Furthermore, plant cells may develop physical barriers that inhibit the spread of pathogens and reduce their ability to cause damage [194,195]. For example, a study found *Fusarium solani* f. sp. After *Glomus intraradical* was inoculated, phaseoli genomic DNA, quantified using quantitative RT-PCR, significantly decreased in the mycorrhizosphere, microsphere, and bulk soil [196]. To increase crop yield, reduce soil erosion, maintain soil fertility, and limit pesticide use, scientists can develop better agronomic practices by understanding the interactions between mycorrhizae, other microorganisms, and plants [197]. Further, understanding the role of mycorrhizae in disease resistance can assist farmers in protecting their crops from soil-borne pathogens and reduce chemical treatment use [198]. A mycorrhiza is a beneficial fungus that forms a symbiotic relationship with plants’ roots to help the plants get more nutrients from the soil and resist pathogens. By understanding how mycorrhizae work, farmers can use them to protect their crops from diseases without relying on potentially harmful chemical treatments. Additionally, certain mycorrhizae can produce safer biocides for humans and the environment [199].

### 2.3. AM Fungi Play an Essential Role in Increasing Crops’ Productivity Maintenance of Soil Health

By doing so, the soil is better able to sustain its structure and fertility over time, providing a stable foundation for various plants and animals [200]. These accumulations are critical for developing and conserving a microporous, water-permeable soil shape, essential for disintegration obstruction and practical supplement cycling [201]. Chemicals and phosphate fertilizers are widely used, which results in pollution problems and health dangers. Therefore, utilizing AMF is energized in horticulture. Taking advantage of Mycorrhiza parasites is not generally simple since it is still impossible to produce a lot of AMF on a large scale in the field [202]. Aside from the results of compost utility on arbuscular mycorrhiza fungi, different techniques such as yield pivot, negligible development, monoculture, culturing, regular changes, and alertness of biocides impact the AMF [203]. Mycorrhiza’s advantageous interaction plays a vital function in the tropics’ horticultural vegetation because, in the tropical locale, there is a phosphorous deficiency in the soil [204]. They do not consume 75% of the phosphorus but get converted to forms inaccessible to plants.

**Figure 2:**
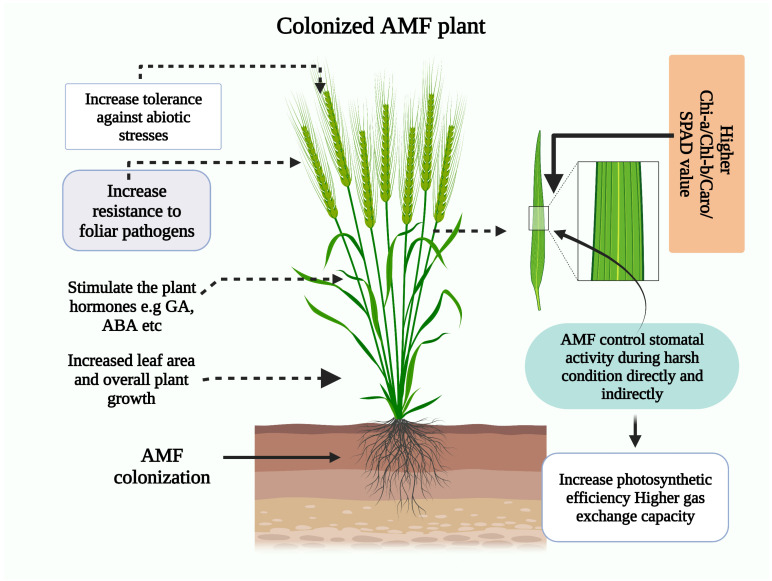
Role of AM fungi colonization in the productivity maintenance of soil health.

Crop plants’ reliance on AMF for nutrient uptake is influenced by root factors, surface area, the quantity and length of root hairs, the rate of growth, and their reactions to soil circumstances and exudations. Smith and Read explained that yields like corn (*Zea mays* L.) and flax (*Linum usitatissiumum*) depend incredibly on arbuscular mycorrhiza fungus to fulfil their advanced demands of phosphorous [205,206]. Mycorrhizal fungi establish a symbiotic relationship with plants, enabling them to enhance their nutrient uptake from the soil. This mutually beneficial association allows plants to access more essential nutrients, including phosphorus and nitrogen. Arbuscular mycorrhizal fungi (AMF) employ various mechanisms to enhance stress tolerance and promote the growth of medicinal and aromatic plants. Arbuscular mycorrhizal fungi (AMF) are crucial in triggering plant responses to abiotic stress. These responses occur at various morphological, physiological, and molecular levels. The purpose of these responses is to help plants cope with the harmful effects of abiotic stress. The symbiosis between arbuscular mycorrhizal fungi and plants has enhanced water and nutrient acquisition, improving plant growth and increasing tolerance to abiotic stress. The symbol “+” is used to indicate a positive effect. EOs refers to essential oils, SOD stands for superoxide dismutase, POD stands for peroxidase, APX stands for ascorbate peroxidase, H_2_O_2_ represents hydrogen peroxide, MDA stands for malondialdehyde, and P represents phosphorus. The relationship depicted in Figure 2 plays a crucial role in promoting the growth and general health of the plant [190].

### 2.4. Role of AMF in the Ecosystem and Mitigation of Environmental Stresses

**Figure 3:**
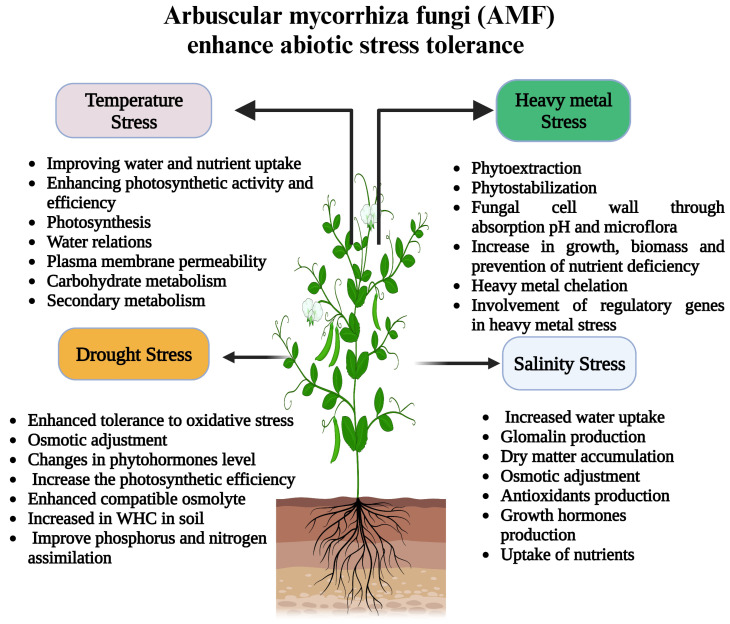
Schematic representation of the different mechanisms imparting abiotic stress tolerance in plants by arbuscular mycorrhiza fungi (AMF).

It is instructive, for instance, to rotate corn and soybean crops to improve soil fertility. Since corn takes up more nitrogen from the soil than soybeans, planting soybeans after corn helps replenish the soil’s nitrogen supply by adding nitrogen [207]. The ‘rotation impact’ situation cannot be defined through nutritional outcomes. Other features containing arbuscular mycorrhiza fungi might also be crucial in fulfilling yield turns, as shown in Figure 3 [208]. Non-mycorrhizal fungi are a reservoir for the soil inoculum, storing and preserving the beneficial microbes. When mycorrhizal plants colonize the soil, they can help to increase the rate at which the beneficial microbes are spread through the soil, thereby increasing the productivity of the soil [209]. A blast in the colonization of AMF also developed in maize came about after sunflower (*Helianthus annuus*, mycorrhizal) when contrasted with corn after mustard (non-mycorrhiza) [210]. This pivot shows the importance of non-ectomycorrhizal vegetation in lowering the rate of AMF colonization among the plants around it. Unlike the colonization of corn after the species alfalfa (*Medicago sativa* L.) and brome grass (*Bromus* spp.), which are mycorrhizal hosts, corn colonization is not an ectomycorrhizal process. Instead, the colonization of corn (*Zea mays* L.) after canola (*Brassica napus* L.) is a non-mycorrhizal host. There was a significant reduction in corn colonization after canola planting for 62 days; however, there was a significant increase in corn colonization similar to that of an AMF host species. In light of these findings, it is possible to increase AMF populations and reverse the inhibitory effects of non-mycorrhizal crops by following up with mycorrhizal crops [211].

The advantages of arbuscular mycorrhiza fungi are significant in frameworks wherein phosphorous inside the dirt is economical [212,213]. Plant tissue phosphorus will rise along with the amount of available phosphorous, and the amount of carbon the plant invests in mycorrhiza is not necessarily profitable for the plant [214]. Support of beneficial mycorrhizal interaction can enhance phosphorous uptake, further developing yield ability without starter P fertilizer spray [215]. AMF additionally plays a critical function in hit reforestation, and numerous reviews of the multiplied foundation of many woodland seedlings inside the field, similar to *Quercus rubra* [216]. A review plan on the foundation of *Desmoncus orthacanthos* and vaccination of AM parasites brought about a triple blast in the endurance of seedlings in the field. Although it is conceivable that different networks of arbuscular mycorrhizal fungus could provide a range of environmental benefits, more research on range/characteristic correlations is necessary to respond to this inquiry [217]. AMF parasitic species/confines can exhibit an unmistakable physiological range.

In contrast, choice or reproduction for plant assortments beneath excessive mineral situations that disregard harmonious movement may result in the era, i.e., kinds of plants that might be less or non-responsive to mycorrhizal [218]. The earliest mycorrhizal morphology combination of the plant and contagious tissues will probably be considered in both companions via simple genetic and ametabolous programs endured over the ages [219]. However, plants have evolved significantly, reaching about 260,000 species that can often be attributed to biological niches or environments that have been described [66]. In evaluation, Glomeromycotan seems to have persisted fantastically unaltered for a considerable number of years, which is a state of affairs that has been explained due to morphological balance [220]; also, something like two hundred transform types of these parasites are known to present daily [221]. A comparable reduction of the cooperative movement because of choice has been located for soya beans [222]. Under low-entry conditions, such adverse effects on the symbiotic feature likely to be neglected in high-input agriculture will be significant. In this context, comparing types conventionally bred with those acclimated to low-input environments will help to light the potential negative influence of breeding on AM characteristics [223].

In any case, it must be noted that no possibility exists where the capacity of a host plant to shape AM via standard choice or rearing exercises has vanished [224]. As such, it is essential to monitor the phosphate levels in the surrounding environment to determine the impact of the fungus on the host plant [225]. Based totally on the finding that plant range impacts the AM fungal range, [226] indicates that long-term monocropping may additionally hurt the AM contagious variety even though it might very well be challenging for each situation to split direct results from the components of complete agrarian control, for example, excessive-supplement, pesticide input, and soil aggravation [227].

#### 2.4.1. Wasteland Salvage

Wastelands can be restored using AMF, which can remarkably heal disturbed fields. Inoculating wastelands with AM fungus can improve the development and endurance of appropriate revegetation [228]. Colonization with AMF may significantly impact the host plant’s growing take-up of soil phosphorus [229]. By utilizing AMF, plant development in badlands can be accelerated. According to specific theories, many plants may also need mycorrhizal contamination to survive on disturbed land. The soil mycelium’s contribution to the absorbent surface area enables substantially better phosphorus uptake. The soil mycelium’s contribution to the porous surface area promotes substantially better phosphorus uptake.

Additionally, host growth is boosted, especially in soils low in phosphorus [72,187]. AM fungi were convincingly proven to enhance the revegetation of coal ruins, strip mines, squander regions, street destinations, and upset regions [230,231]. The expansion of AMF has a dietary benefit to related plants, supplying viable resistance from lower pH, weighty foil toxicants, and excessive temperature [232,233]. The appearance and utilization of AMF have significantly expanded rehabilitation success in these moisture-deficient zones. Pre-inoculating nursery seedlings with suitable mycorrhizal fungus can benefit revegetate disturbed mining grounds [234,235].

#### 2.4.2. Mitigation of Drought Stress

Research has demonstrated that various factors can hurt plant life during drought conditions. One such factor is the insufficient availability of water for the roots, which can lead to decreased rates of transpiration and an elevated level of oxidative stress on the plant. Drought stress harms plant growth due to various factors, such as enzyme activity, ion uptake, and nutrient absorption [236]. Barley, wheat, maize, strawberries, soybeans, and onions are just some crops that have improved drought stress after being treated with AMF. The depth to which a plant’s roots and extra-radical hyphae probe the ground may be related to how well it can tolerate dryness [191].

**Figure 4:**
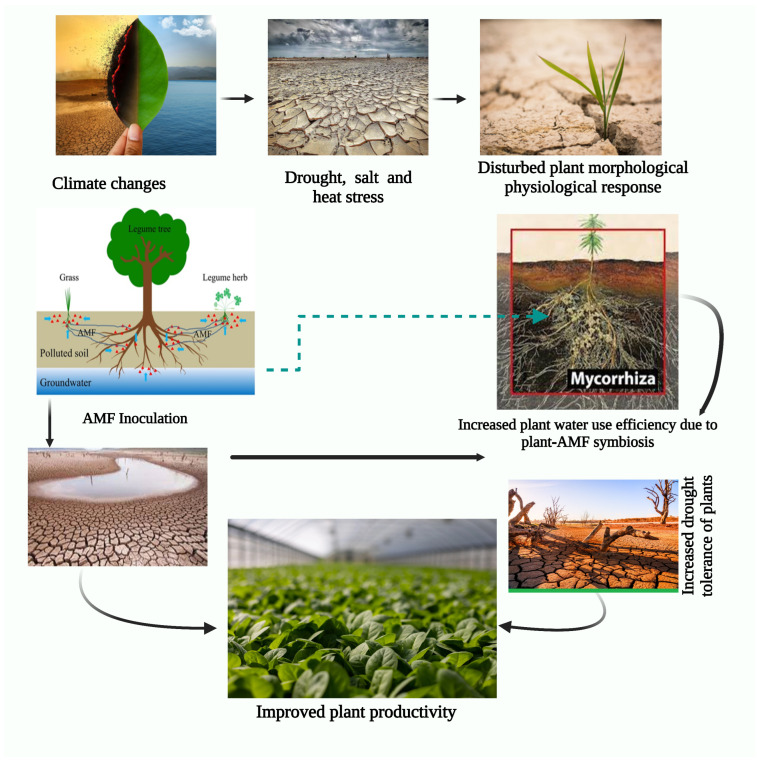
Alleviation of drought stress effects by AMF inoculations and mycorrhizal association.

This means that the symbiotic relationship between the symbiotic organism and the plant can bring about a range of positive effects, such as increased osmotic adjustment, which allows plants better to regulate the amount of water in their cells, increased proline accumulation, which helps to protect the plants from various environmental stressors, and increased glutathione levels, which provide the plants with a source of antioxidants [237,238]. This is because beneficial fungi provide an extra source of nutrients, moisture, and protection from plant diseases, allowing them to withstand drought conditions better. In addition, the root systems of plants that AMF colonizes are often larger and more branched, increasing the surface area for absorbing water and nutrients [239]. This study shows that the beneficial relationship between AMF and plants can increase their overall performance as AMF can stimulate various physiological processes in plants [240]. Water and nutrients can be efficiently absorbed as part of these processes, which can help optimize photosynthesis and other metabolic processes, thereby maximizing their productivity. It has recently been demonstrated that the antioxidant system in C3 plants (*Leymus chinensis*) and C4 plants (*Hemarthria altissima*) can be upregulated by AMF through activity in the AMF-mediated pathway [241]. H_2_O pressure is a chief agricultural limitation inside the semi-dry tropics. It is widely recognized to have a dreadful effect knob capability [242]. It prevents the process of photosynthesis and messes with the fragile oxygen-management system in knobs. The last option is crucial for the energetic obsession with nitrogen. AMF beneficial interaction can safeguard plants from water pressure’s negative consequences, as shown in Figure 4 [136,243]. Aalipour et al. (2020) detailed that immunization with native inoculants improved leaf and root development and avoided normal expansion in root-to-shoot and root-weight proportions typically located below phosphorus-lacking and water pressure conditions in peanuts. AMF works on consuming minerals such as nitrogen and phosphorous in water-focused states [136].

Insufficient soil water is communicated to the shoots via unpressurized chemical indicators. With the help of the transpiration system, this is transmitted from the roots’ drying out to the aerial shoots [244]. The reaction is indicated by employing the leaves regarding little growth and reduced stomata conductance. Arbuscular mycorrhiza fungi change the non-water-driven root to shoot, signaling soil dryness via disposing of the leaf reaction [245]. Greater extremist hyphae of AMF expand the roots’ absorbent surface region [246,247], lowering the impedance to water take-up. AMF’s part in reducing plant life’s water stress has been examined, and drought resistance appears higher. Often, a growing dependence on AMF for nutrient intake can be seen. As a result, AMF helps to reduce water pressure issues [248].

#### 2.4.3. Bioremediation

Heavy metal pollution decreases the activity of microbial processes and soil microorganisms. High heavy metal toxicity to soil microorganisms and microbiological processes linked to long-haul impacts inside the soil are crucial facts [249,250]. Along with AMF, all microorganisms display protection from weighty metals by demonstrating either “toleration”, where the life form may continue to function in the existence of elevated inner metal fixations, or “aversion”, where the organism can confine metal take-up [251,252]. Phytoremediation, using plants to remove toxic metals from the soil, is gaining popularity as a feasible and environmentally friendly method of cleaning polluted land [118,253]. According to reports, AMF has developed strategies that involve immobilizing metallic compounds, precipitating particles of phosphate in the ground, adsorbing chitin within the cell wall made up of fungus, and, ultimately, chelating heavy metals inside the AMF [254]. These strategies could reduce heavy metallic dangers in blended subculture systems and, as a result, from the natural pecking orders [255]. Roots of plants having mycorrhiza colonization can lessen the movement of weighty foils to shoots by restricting the heavy foil to the cell wall of contagious threads in the source. Along these lines, mycorrhiza can assist higher crops with adjusting to their current circumstance and getting by in polluted regions. Arbuscular mycorrhiza is effective for protecting plants from heavy soil foil, as evidenced by the synergistic effects of saprophytic parasites, such as *Fusarium concolor* and *Trichoderma koningii*, on root colonization of plants through AMF [256]. A researcher suggested that the AM hyphae may function as metal filters in the plant by encasing the potentially dangerous substances in the polyphosphate granules. Metal toxicity can affect many AM fungal lines. Different AM fungus lines are susceptible to metal poisoning. AMF exertions that colonize a plant thus influence its resistance to death [257]. Because the fungus produces so many outer hyphae, they may be concerned that they will capture the metal and protect the plant. This would rely on the ecological modifications performed by the AM as a result of the presence of dangerous metals. The mycorrhizal parasite *Glomus caledonium* seems to be a good choice for bioremediating soil polluted with massive steel [258,259].

#### 2.4.4. AMF-Induced Changes in the Host to Deal with HMs

Fast-growing hyphae that can thrive under challenging environmental conditions, such as metal toxicity, help the host plants form symbiotic relationships [208,222,260]. Since AMFs can strengthen the defence mechanism of AMF-mediated plants, it is widely considered that they support plant establishment in soils contaminated with heavy metals. The accumulation of heavy metals in crops, fruits, vegetables, and soils can lead to several health problems. During symbiosis, AMF can trigger a wide range of changes in the host plant, aiming to equip it better to handle its dangerous surroundings. AMF indirectly induces plant tolerance by increasing water and nutrient intake, increasing shoot biomass, and altering root architecture. Through metal adsorption on the fungal surface and glomalin immobilization in the soil, arbuscular mycorrhizal fungi can remove toxic heavy metals from polluted environments [1,24,35,60,261]. One of the tolerance mechanisms AMF hyphae uses, which is the direct engagement of fungi to provide a physical barrier to HMs in the entry into plants, is the chelation and sequestration of HMs by their fungal structure. AMF assisting plant remediation is an environmentally friendly, cost-effective, and stable green remediation technology. It has much potential for restoring the ecological balance of heavy-metal-contaminated soil [17]. Inoculation with mycorrhizal fungus, particularly Rhizophagus aggregates, causes glomalin production, drastically lowering branches’ Cd, Pb, Zn, and Cu concentrations [253]. It alleviates Zn stress by enhancing food uptake and modulating Zn uptake at the gene transcription level. In addition, a large body of research indicates that heavy metal-induced ultrastructure damage in host plant cells can be considerably mitigated by AMF inoculation [211]. Kuhn found that the mycorrhizal fungus’ cytoplasm has higher concentrations of Cd, Ti, and Ba than the fern host cells by electron energy loss spectroscopy; he termed this combination the “filtering” mechanism. Inoculation with *Acaulospora morrowiae*, *Gigaspora albida*, and *Rhizophagus clarus* (AMF) significantly alleviated symptoms [7]. The ultrastructure of *Leucaena leucocephala* (Lam.) leaf and root cells can be seen under stress [118]. Subcellular Cd uptake and dispersion is how AMF colonization makes *Phragmites australis* (Cav.) Trin. ex Steud. more Cd tolerant, as discovered by Huang et al. Cd absorbed by AMF was shown to be intraradically immobilized, shedding light on the direct absorption and transport by AMF [7,69,262]. It demonstrated the geographical distribution of Cd in mycorrhiza after Cd absorption by fungi. However, it is unknown if AMF may mitigate ultrastructural alterations of woody plant roots caused by Cd toxicity or what concentration of Cd is hazardous to mycorrhizal eucalyptus at the cellular level [7,186].

#### 2.4.5. Temperature (High and Low)

**Figure 5:**
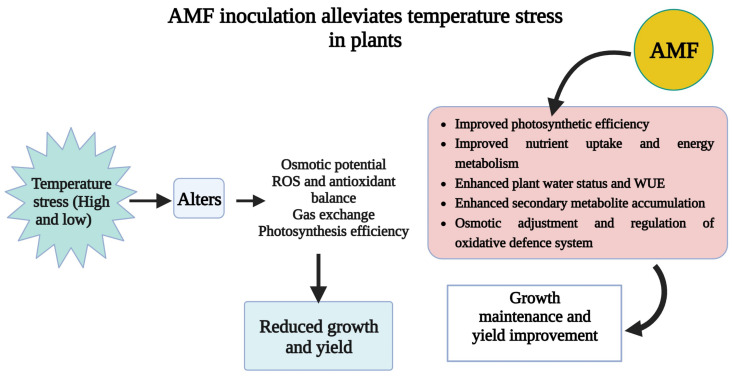
AMF inoculation alleviates temperature stress in plants.

Plant community responses may depend on AMF interactions as soil temperatures rise for sustainable yield and output. Heat stress has a significant impact on plant growth and development by causing the following effects: (i) loss of plant vigor and suppression of seed germination; (ii) slowing of growth rate; (iii) decreased biomass production; (iv) wilting and burning of leaves and reproductive organs; (v) abscission and senescence of leaves; (vi) damage and discolouration of fruit; (vii) decrease in yield and cell death; and (viii) reduce plant height and branches [26,263]. There has been much research on the correlation between AMF *Glomus fasciculatum* and plant growth and development, and it has been found that inoculating plants with AMF lead to favorable changes in growth when exposed to high temperatures (Figure 5; Table 1).

AMF can increase plant tolerance to cold stress. Moreover, most reports state that plants inoculated with low-temperature AMF grow better than non-AMF-inoculated plants. AMF supports plants in combating cold stress and eventually improves plant development. Moreover, AMF can retain moisture in the host plant, increase plant secondary metabolites, strengthen the plant immune system, and increase the protein content to support the plants in combating cold stress conditions [59,223,244,264]. For example, AMF-inoculated plants enhanced water conservation capacity and efficiency during cold stress. A symbiotic AMF relationship improves water and plant relationships and increases gas exchange potential and osmotic adjustment. AMF improves chlorophyll synthesis, significantly improving the concentrations of various metabolites in plants subjected to cold stress conditions. The role of AMF during cold stress has also been reported to alter the protein content in tomatoes and other vegetables [45,51,218].

#### 2.4.6. Salt Strain

In addition to posing a severe threat to food security worldwide, soil salinization poses a serious environmental problem. Furthermore, it produces excessive amounts of reactive oxygen species, which harm plant growth. The laboratory has extensively studied how salt stress impacts plant growth. In the context of mitigating salt stress-induced oxidative damage, recent research by Afrangan et al. [263] highlighted the regulatory roles of *Glomus versiforme* and *Micrococcus yunnanensis* in maintaining a redox state and ion homeostasis, as demonstrated in *Brassica napus* L. crops. This study’s results showed that they negatively impact plant growth by inhibiting vegetative growth and net assimilation, which lowers yield productivity. Besides improving crop productivity in salinized soils, AMF has been applied judiciously to mitigate the adverse effects of salinity on plants [265]. It has been demonstrated in several research studies that AMF increases plant growth and yield when plants are subjected to salinity stress. According to Ait-El-Mokhtar et al. (2019), AMF symbiosis is beneficial under saline regimes [266,267], and AMF significantly reduces harmful effects on photosynthesis in salinity stress. Inoculating mycorrhizal plants significantly enhanced the photosynthetic rate, gas exchange traits, chlorophyll content, and water use efficiency of *Ocimum basilicum* L. through mycorrhizal inoculated plants. As a result of AMF inoculation [268], it has been found that *allium sativum* plants grow better under saline conditions, particularly in terms of leaf area index and fresh and dry biomass [269]. Mycorrhizal inoculation under moderately salinized conditions leads to significant increases in fresh and dry weights and the N concentrations of shoots and roots, which are significantly higher [270]. Growth-promoting factor levels can be efficiently managed by inoculating AMF to the patient. Improvements in cytokinin concentration led to a noticeably higher photosynthetic flux when plants were subjected to salt stress [271,272]. AMF-mediated growth enhancement under salt stress may be caused by a change in the polyamine pool [273]. Increased strigolactone dramatically reduced the effects of salt on lettuce plants that had received AMF treatment [274]. Previous studies show that plants colonized with AMF exhibit reduced oxidative stress by reducing lipid membrane peroxidation under salt stress [272,275]. In addition, inoculation of AMF increased the accumulation of organic acids in plants subjected to saline stress, increasing osmoregulation. It was shown that plants grown in saline soil produce more organic acids and that AMF increases betaine production in maize plants. This suggests that AMF indirectly affects plant osmol by directly affecting its osmoregulation under salinity stress [267]. AMF can modify plant properties, including physiological and morphological, using plants that cope with the strain [265,276]. Arbuscular mycorrhizal fungi promote a higher survival rate of plant life under stressful circumstances via an increased intake of minerals, especially phosphorous, copper, zinc, and water [28]. They are the most resistant to poor conditions brought about by soil or weather-related harmful elements. This response provides a concise overview of the role played by arbuscular mycorrhizal (AM) fungi in mitigating plant stress resulting from various factors such as grazing, salt, metallic pollution, and drought [119,167].

Salinization of soil is a severe issue that regularly grows in some areas of the world, specifically in arid and semi-arid locations [186,277]. Salinity stress may be reduced with the help of AMF. Physiological stresses are placed on plants growing in saline soils. The harmful effects of particular ions, including sodium and calcium, found in salty soils alter the framework of ions and some other large molecules, harm cellular organelles, impair the process of respiration and photosynthesis, obstruct protein synthesis, and cause ion deficits [278,279]. Even though many halophytic plants have a relatively low mycorrhiza affinity, AMF naturally occurs in salt conditions. In moderately salty soils, AMF can guard a few non-halophytic plants against yield losses. Possible processes include increasing plant nutrients, promoting root development [280], and manufacturing plant polyols in the mycorrhiza plant. Arbuscular mycorrhiza fungus aids in the enhanced addition of P, N, and other development-upgrading vitamins, which can also be a helping hand for the expected development of flora in the salted plant [281].

#### 2.4.7. AMF and Combined Abiotic Stresses

It is generally agreed that AMF can reduce the effects of stressors like drought, salinity, temperature, nutrients, and heavy metals, alone or in combination. For instance, plants subjected to both drought and salinity see an increase in the creation of reactive oxygen species, which can harm plants [35,136,276]. Enzymes such as catalase, peroxidase, glutathione reductase, and superoxide dismutase detoxify reactive oxygen species (ROS). Biomass output, leaf water relations, stomatal conductance, and Fv/Fm were all enhanced in tomato plants inoculated with *Scolecobasidium con-striatum* after they were subjected to a combination of drought and salinity [34,64,249,282]. As a result, AMFs are crucial for maximizing plant growth and productivity while under duress. Few studies have indicated that AMFs can mitigate the destructive effects of multiple stressors acting in concert. AMF symbiosis protects plants from a variety of abiotic stresses through processes such as enhanced photosynthetic rate, mineral nutrient intake and accumulation, Osmo protectant accumulation, antioxidant enzyme activity modulation, and adjustments to the rhizosphere ecology [20,197,283]. In several experiments, osmotic stress conditions, such as deficiency irrigation or salinity, have been demonstrated to increase the nutritional quality of AMF plants. Similarities among tolerance mechanisms are possible in response to AMF-mediated combined stress adaptations [37,61,67,284]. It is hypothesized that a common mechanism generated by various stresses is AMF-mediated changes in phytohormone profile, mineral absorption and assimilation, buildup of suitable osmolytes and secondary metabolites, and up-regulation of the antioxidant system [36,220,226,285]. However, stress type and the AMF species involved can cause significant changes in the expression of some proteins and the processes that plants use to compartmentalize and sequester harmful ions. Root features like hydraulic conductivities can be modified to significantly raise osmotic stress tolerance [221,235,286,287]. By modifying gas exchange features and the amounts of some essential metabolites, the researchers found that AMF protected castor beans from saline stress. It is possible that AMF’s unique properties could improve the crops’ nutraceutical quality, making them more useful for human consumption. This could have significant agronomic importance for cultivating and administering various crops. However, in-depth research is needed to determine AMF’s part in mitigating the outcomes of several stressors [59,215,223,224,244,264].

## 3. Importance of AMF in Soil Fertility

Plants and trees benefit from the symbiotic relationship between mycorrhizae and their roots. These fungi form a network of filaments around the roots, which increases the surface area of the root and provides access to a much larger area of soil. This increases the ability of the root system to take up essential minerals, such as phosphorus, copper, and zinc, which are typically less mobile in the soil [288]. Furthermore, mycorrhizae can help crops establish themselves in poor soil conditions and improve their nutrient uptake. Mycorrhizae are symbiotic fungi that collaborate with plant roots to provide essential nutrients in return for carbon fixed through photosynthetic processes. Because the plant roots remain immobile in the soil, they would not have as much access to nutrients without this relationship between the fungus and the roots [289]. Moreover, fungi help plants to establish themselves in poor soil conditions and improve their nutrient uptake, leading to better crop yields and reducing the need for fertilizers. This is because the microorganisms use the root exudates to obtain nutrients for their growth and, in doing so, create a nutrient-rich environment for the plants.

Additionally, the microorganisms release compounds with antibacterial and antifungal properties, which help to protect the plant roots from disease-causing organisms [290]. They also produce compounds that bind to harmful chemicals and help to reduce their toxicity. Numerous studies have demonstrated that AMF may overcome nutritional challenges to plant development by enhancing nitrogen uptake [291]. Most studies have focused on phosphorus intake, but mycorrhizal has also been linked to the absorption of various essential vitamins. Mycorrhizal flora is exceptional in absorbing inorganic nutrients because the fungal hyphae provide a large surface area for mineral uptake compared to uninfected roots [72,292]. Because of the growth of fungal mycelium via soil, it scavenges for nutrients and vitamins and can touch roots that are not infected, often from different host varieties [264]. Compared to roots, greater-radical mycelium is smaller and can penetrate fewer crystalline minerals, aggregates, and organic materials that roots can access alone. By the secretion of enzymes, inaccessible types of phosphate can be solubilized [293]. Phosphorus is the primary nutrient needed by plants in relatively high concentrations. It is essential for all biological processes, including energy transfer via forming phosphate esters rich in energy. It plays a crucial role in developing macromolecules like nucleotides, sugar phosphates, and phospholipids [294,295]. One of the numerous vital advantages of mycorrhizal is the expansion in P consumption via the plant. The overall strategy of phosphorous uptake comprises three sub-methods: (i) assimilation through the dirt via AMF hyphae, (ii) movement along the hyphae from outside to inside (root cortex) mycelia, and (iii) the exchange of P to cortical cells of the root [287]. For instance, when legumes are planted in combination with grains, legumes can take up phosphorus from the soil, which is then released into the soil when the legumes are harvested, thus providing the grain crop with phosphorus [296]. Roots absorb P more quickly than ions diffuse to their absorption surfaces. This brings on the phosphate depletion zone around the roots.

**Figure 6:**
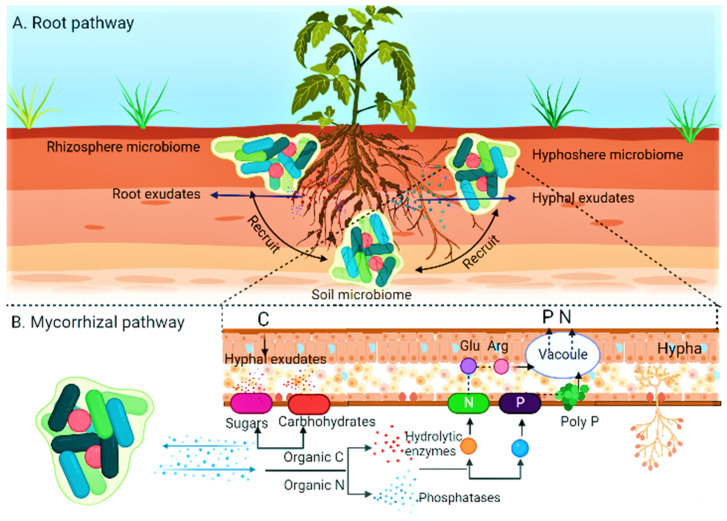
The symbiotic interaction between plant roots and mycorrhizal fungi improves nutrient uptake and crop production. Fungi release chemicals with antibacterial and antifungal effects and increase the available surface area for mineral absorption.

To span the quarter of nutrient loss, the extensive extra metrical hyphae of AMF extend several centimeters into the soil, as shown in Figure 6. The plant can benefit from microhabitats outside the mineral-poor location, which, without roots and hair, cannot survive [283]. Nitrogen is required to form amino acids, purines, and pyrimidines and is, in a roundabout way, engaged with synthesizing nucleic acid and protein [261]. In addition, AMF-associated plants are known to produce root exudates that contain nitrogen-rich organic compounds, further contributing to the increased nitrogen concentration observed in shoots [154]. The AMF hyphae typically draw nitrogen from the soil and move it to the plants. They include nitrogen reductase, which modifies nitrogen patterns in soil, and enzymes that degrade organic nitrogen [297]. AM better the development, nodulation, and fixation of nitrogen in a legume–*Rhizobium* association. In addition, they consume NH4+ with ease from the soil, which shapes the more significant part of accessible nitrogen in numerous standard biological systems [298]. AMF has a negligible impact on obtaining nitrogen through vegetation in land where nitrate is a predominant nitrogen originator [270].

According to previous studies, the association of mycorrhizae provides more than 50% of the N that plants need. Mycorrhizal corn’s roots and shoots and mycorrhizal inoculation boosted the exercises of nitrate reductase, glutamine synthetase, and glutamine synthase *Zea mays* L. [299]. An ammonia transporter within plants is activated when AMF is present, suggesting nitrogen is transferred like phosphorus [300]. Many mycorrhizal fungi can access inorganic forms of N and P. Still, some generated proteases also transfer dissolvable amino compounds into the root via the hyphal framework when they live in the litter [301]. According to recent research, the Glomus bacteria can move Glycine and glutamine into wheat [282]. It is believed that plant physiology is altered to reduce the pressure reaction caused by the AMF association during dry soil seasons or to enhance the pressure-driven radioactivity of water uptake by the roots to decrease the pressure reaction [302]. It demonstrates how the mycelia network spreads in the dirt to find nutrients and water. The advanced nutrition of phosphorous and mycorrhizal colonization by AMF can move plants’ resilience to drought by changing cell membrane permeability to water [263]. Below situations of dry season pressure, AMF exerts its effect on developing the happening amount and decreasing stomata opposition or changing the equilibrium of plant chemicals [303]. The improvement in leaf turgor and water capacity caused by AMF inoculation also enhances root length and intensity. This change in leaf elasticity may also affect water relations and the vegetation’s ability to withstand drought. The increased conveyance of the retaining hyphal community, the more favorable geometry of hyphae in contrast with roots, the expanded ground location, the quicker augmentation rate, the enhanced good toughness, the chemical changes in the soil’s rhizosphere, the changed rhizosphere microbial populace, the uptake energy, the high water is driven conductivities, and the decreased transportation quotes are likely causes for the high water and supplement uptake quotes via mycorrhizal plants [206]. Because of atmospheric disturbances, the soil temperature, humidity, and nutrient availability can change. By reducing the amount of nutrients and water available, these changes can adversely affect the activity of AMF. As a result of these changes, soil aggregation and conservation may decrease, leading to erosion [304]. A combination of Glomalin and other components can create a soil structure by binding particles together and forming aggregates that can resist erosion. AMF hyphae can also increase nutrient availability by taking minerals from the soil’s surroundings, thus making the soil more fertile and nutrient-rich. Mycorrhizal fungi form a network of hyphae around plant roots as they grow.

In addition to increasing the plant’s ability to absorb water and nutrients, this network also provides a strong barrier against disease. As well as releasing exudates into the soil, the fungi increase microbial activity, improving soil fertility and structure [240]. There are many benefits gained from this process for both the soil and the plant, such as the facilitation of nutrients being absorbed by the plant, the increase in aeration of the soil, improved water retention, and suppression of disease-causing pathogens due to the presence of mycorrhizal hyphae [305]. This organization is essential for the proper functioning of ecosystems because it plays a role in nutrient cycling and water retention and provides a protective barrier against soil erosion [306]. The sticky substance Glomalin, found in healthy soil, binds soil particles together with the help of fungi. This binding action allows for the efficient absorption of water and nutrients by plants. As a result of the binding action, soil structures are stabilized, and more water and nutrients are retained [294,306]. In a capability environment containing AM capabilities, an intricate, ramifying mycelium community’s root shape and growth were altered (binding action and improvement of soil structure) regarding soil growth, plant adhesion, and soil balance. Plants’ increased uptake of water and minerals leads to improved plant growth and development, while fertilizer needs to be decreased [284]. Using protection, the plants can absorb more nutrients, fight disease, and retain water more effectively, creating a more hospitable environment. The soil balance is also enhanced, providing better conditions for the growth of beneficial microorganisms like fungi, which can protect plants from biotic stressors [7]. In addition to being able to absorb more nutrients, fight diseases more effectively, and retain more water, plants can create a more hospitable environment by using protection [262]. Because of this, this process enhances the soil balance, which creates a climate conducive to the growth of beneficial microorganisms such as fungi, which can protect plants from biotic stresses [307]. Monoculture editing, ploughing, or fertilization adversely affect the number and range of arbuscular mycorrhiza fungi in soil. The reduction of parasitic biofuels compromises soil stability and increases the risk of soil disintegration [10]. In addition to decreasing the fertile soil available for farming, soil erosion has depleted essential nutrients for healthy crop growth. This has led to an overall decline in agricultural productivity. Due to this, agricultural production has decreased in the country, resulting in higher prices and decreased food security [308]. As a result of increased phosphorus uptake from the soil, the AM fungus helps increase the amount of phosphate in the soil. This reduces the need for phosphate fertilizer since the soil can provide more phosphorus independently. The AM fungus reduces soil erosion by helping to bind the soil together and keep it in place. In this manner, the soil is less likely to be washed or blown away. The phosphorus mineral also plays an essential role in photosynthesis, respiration, energy transfer, and enzyme activity [309]. Furthermore, it is essential for root development, fruit, and vegetable formation. Additionally, phosphate aids plants in absorbing other essential nutrients from the soil. Since the global population is rapidly expanding and the demand for phosphate is increasing, the mining industry is unable to keep up with the industry’s rapid growth [310]. Furthermore, phosphate reserves are often located in inaccessible or politically unstable areas, increasing the difficulty of exploiting them [264]. However, three-phosphate uptake in developed international locations decreased by 36% between 2000 and 2006, resulting in annual consumption of 0.3 million tons. However, at the same time, the amount of three-phosphate consumed by agricultural nations has increased by 36%, reaching an annual amount of 2.1 million tons [260]. The application of phosphate fertilizers in various ways has contributed significantly to the eutrophication of water bodies, because of which plants have a difficult time developing phosphate absorption efficiency [311]. This limited soil distribution leads to a decrease in the Pi concentration in the soil in the area immediately around the roots, resulting in a Pi depletion zone. This zone limits the availability of Pi uptake by the plant, leading to a decrease in the phosphate (Pi) directly absorbed by the surface [312]. Mycorrhizal fungi form a beneficial relationship with the plant. The fungi absorb essential nutrients from the soil, such as phosphorus and nitrogen, and then pass them on to the plant. This increases the plant’s capacity to absorb these nutrients and can improve growth and health [313]. This proposed solution would reduce the amount of chemical fertilizer needed and could help to reduce the environmental impact of using fertilizers. It could also help reduce the cost of fertilizing for farmers since the cost of using the AM fungus is much lower than traditional fertilizer [152].

## 4. Conclusions

Most study papers have already provided evidence of the positive effect AMF has in enhancing plant growth in conditions considered stressful. As a result, the current material related to the role of AMF has been combined cohesively in this review to understand the symbiotic relationship that AMF has with various plants when exposed to stressful situations. In the past, the AMF has been primarily discussed as a beneficial entity for the uptake of nutrients from the soil. However, in more recent times, it has been depicted that plants inoculated with AMF can effectively combat various environmental cues, such as salinity, drought, nutrient stress, alkali stress, cold stress, and extreme temperatures, and can therefore help increase the yield per hectare of a wide variety of crops and vegetables. Arbuscular mycorrhizal fungi (AMF) can increase the absorption of nutrients such as phosphorus, nitrogen, and zinc and increase the availability of some essential micronutrients. By colonizing the soil, AMF can improve the soil structure and boost the activity of beneficial microorganisms, which can help plants become more resistant to drought and disease. Therefore, enhancing the colonization of AMF in the soil can help to improve crop productivity and yield in the future.

By identifying and improving the characteristics of AMF accessibility, functionality, and climate resilience in new cultivars, farmers can maximize their productivity and increase the sustainability of their production. This will also ensure food production, which is essential for global food security. By integrating this system, we can significantly reduce the amount of energy and artificial input necessary while enhancing the effectiveness of beneficial organisms and the sustainability of agricultural systems. In addition to improving yields, this method can significantly reduce crop losses due to pests, diseases, and extreme weather events since it optimizes using natural resources, such as water and nutrients. Many researchers believe that by administering mycorrhiza to plants, they can absorb more soil nutrients and water. In addition, they will be able to withstand diseases and stressors better since they can absorb more nutrients and water. As a result, reducing fertilizer and other agricultural inputs contributes to enhanced sustainability and cost-effectiveness by concurrently improving crop yields and quality. As well as providing essential nutrients for crop production, such as by adding nitrogen, phosphorus, and potassium to the soil, AMF also retains moisture in the soil, which is crucial to the growth of crops. Aside from these benefits, there are also other benefits, including the reduction of erosion, the reduction of weeds, and the improvement of the soil’s organic matter level. These benefits will, in turn, likely lead to increased agricultural productivity and an improved environment due to all of these benefits. Because of a symbiotic relationship between mycorrhizal fungi and plants, the plants have an easier time accessing nutrients and water. By doing this, the plants can grow faster and healthier and reduce toxic elements in the soil, which may result in higher yields, improved soil quality, and a more stable ecosystem.

## Figures and Tables

**Table 1:** Arbuscular mycorrhizal isolates provide a range of agroecosystem services to important crops among smallholder farmers, and such services have been well documented in numerous recent studies.

AMF Symbionts	Crop Tested	Agroecosystem Service	Conditions	References
*Rhizophagus irregularis* and *Funneliformis mosseae*	Chickpea (*Cicer arietinum* L.)	Enhanced plant mass and superior grain characteristics.	Agriculture land	[24]
A mixture of *Glomus clarum*, *Gigaspora margarita*, and *Acaulospora* sp.	Coffee (*Coffea arabica* L.)	Protection against Cu and Zn toxicity.	Greenhouse	[25]
Indigenous AMF consortium: *G. etunicatum*, *G. fasciculatum*, *Glomus mosseae*, *G. intraradices*, and *Scutellospora* sp.	Green pepper (*Capsicum annuum* L.), parsley, and tomato (*Solanum lycopersicum* L.)	Elevated crop yields and superior plant vitality.	Greenhouse and field	[26]
G. mosseae Consortium: G. intraradices, G. aggregatum/intraradices, *Entrophospora infrequens Acaulospora trappei*, and *Glomus* sp.	Lettuce (*Lactuca sativa* L.) and Onion (*Allium cepa* L.)	Tolerance to saline environments.	Greenhouse	[27]
*G. fasciculatum*, *G. etunicatum*, *G. clarum* and *G. versiforme*	Long pepper (*Piper longum* L.)	Superior growth attributes.	Garden centre and field	[28]
*G. aggregatum*, *Consortium: R. intraradices*, *G. viscosum*, *G. etunicatum*, and *G. claroideum*	Maize (*Zea Mays* L.)	Optimal crop growth, output, and grain attributes.	Arena	[29]
Native AMF	Maize (*Z. mays* L.)	Augmented absorption of K, Ca, and Mg.	Arena	[30]
*G. mosseae* and *G. intraradices*	Lucerne (*Medicago sativa* L.)	It boosted GRSP and better soil cohesiveness.	Greenhouse	[31]
*G. etunicatum*, *G. mosseae*, *G. fasciculatum*, and *Gigaspora margarita*	Pepper (*C. annuum)*	Growth promotion and mitigation of Phytophthora infections.	Pot, greenhouse, and field	[32]
*Glomus clarum*	Pepper (*C. annuum*)	Augmented fruit production in saline-rich conditions.	Glasshouse	[33]
*On-farm produced G. intraradices*	Potato (*Solanum tuberosum* L.)	Bigger harvests.	Field	[34]
*G. etunicatum*	Soybean (*Glycine max* (L.) Merr.)	It helps to elevate growth performance under saline circumstances.	Greenhouse	[35]
*G. mosseae*	Soybean (*G. max*) and Lentil (Lens culinaris Medic)	Enhanced absorption of Zn.	Greenhouse	[36]
*G. intraradices*, *G. mosseae*, and *G. etunicatum*	Strawberry (*Fragaria × ananassa* Duch.)	Top-tier quality.	Field	[37,38]
*G. intraradices*	Strawberry (*F. × ananassa*)	Boosted fruit attributes.	Glasshouse	[39]
*G. mosseae* and *G. hoi*	Sunflower (*Helianthus annuus* L.)	It helps to promote advanced agricultural yields.	Glasshouse	[32]
*G. mosseae*, *G. intraradices*, and *G. coronatum*	Sunflower (*H. annuus*)	Tackling wildflowers and optimizing phosphorus absorption.	Greenhouse	[39]
*Native AMF inoculum (consortium)*	Tomato (*Solanum lycopersicum* L.)	Modifying plant responses to zinc inclusions.	Environment chamber	[40]
*G. intraradices*	Tomato (*S. lycopersicum*)	Addressing the root disease caused by Alternaria solani.	Climate chamber	[41]
*G. intraradices*	Tomato (*S. lycopersicum*)	It enhanced cultivation, processing, and fruit quality in waterlogged conditions.	Field	[42]
*G. mosseae*	Tomato (*S. lycopersicum*)	Addressing issues from root-knot nematode Meloidogyne incognita.	Greenhouse	[43]
*G. intraradices*	Tomato (*S. lycopersicum*)	Superior resilience to saline conditions.	Glasshouse	[44]
*G. intraradices*	Tomato (*S. lycopersicum*) and Onion (*A. cepa*)	Advanced harvest results.	Field	[29]
*G. intraradices*	Tomato (*S. lycopersicum*)	Dealing with the deceptive root-knot nematode Nacobbus aberrans.	Greenhouse	[45]
*G. intraradices*	Tomato (*S. lycopersicum*)	Augmented growth, flowering, and yield.	Field	[46]
*G. mosseae*	Tomato *(S. lycopersicum*)	Boosted growth cycle, flowering stage, and fruit formation.	Growth chamber	[47]
*G. mosseae* and *G. intraradices isolates* and *native AMF inoculum (consortium)*	Clover (*Trifolium alexandrinum* L.) and maize (*Z. mays* L.)	Superior agricultural output.	Field	[26]
*G. etunicatum* and *G. mosseae*	Wheat (*Triticum aestivum* L.) and Brinjal (*Solanum melongena*)	It helps to enhance plant growth, yield, and nutrient absorption.	Field	[48]
*G. mosseae*, *G. hoi*, *G. etunicatum*, *A. scrobiculata*, and *A. spinosa*	Yam (*Dioscorea rotundata*)	It helps to boost root expansion.	Greenhouse	[26]
Consortium of *G. mosseae*, *G. deserticola*, and *A. laevis*	Yam (*D. rotundata* and *D. alata*)	Elevated nutritional content.	Glasshouse	[49]

**Table 2:** Contribution of AMF in helping plants to cope with biotic and abiotic stress.

Host Plant	AMF Strains	Stress	Observed Responses	References
*Solanum lycopersicum*	*Rhizophagus irregularis*	Salinity	Growth hormone promotes plant health, increasing root and shoot weight and improving leaf structure.	[106]
*Leymus chinensis*	*Glomus mosseae*	Salinity	Positive outcomes include elevated phosphorus and nitrogen levels, enhanced seedling weight, and increased plant water content.	[107]
*Cucumis sativus* L.	*Claroideoglomus etunicatum*, *Rhizophagus intraradices*, *Funneliformis mosseae*	Salinity	Enhanced growth, higher antioxidant enzyme activity, elevated proline and phenolic content, and improved uptake of vital mineral elements have been observed. Furthermore, the absorption of sodium ions was reduced.	[108]
*Medicago sativa*	*Glomus mosseae*	Salinity	Among the crucial nutrients for plants, phosphorus (P), nitrogen (N), and potassium (K) play pivotal roles.	[109]
*Glycine max* L. Merrill	*Claroideoglomus etunicatum*, *Rhizophagus intraradices*, *Funneliformis mosseae*	Salinity	Benefits encompass improved plant and root systems, heightened nutrient uptake, minimized lipid peroxidation, and reduced membrane damage.	[27]
*Prunus dulcis x Prunus persica hybrid*	*Rhizophagus intraradices*, *Funneliformis mosseae*	Salinity	Plant growth sees improvements through elevated antioxidant enzymes, increased photosynthetic compounds, soluble sugars, and proline content.	[110]
*Pisum sativum* L.	*Rhizoglomus intraradices*, *Funneliformis mosseae*, *Rhizoglomus fasciculatum*, *Gigaspora* spp.	Salinity	Enhanced biomass, chlorophyll content, nutrient absorption, and accumulation of compatible osmolytes contribute to overall plant well-being.	[111]
*Euonymus maackii* Rupr	*Rhizophagus intraradices*	Salinity	Notable advantages include enhanced photosynthesis, increased nutrient assimilation, and improved antioxidant enzyme activity.	[112]
*Citrullus lanatus* L.	*Glomus mosseae*, *Gigaspora gigantean*	Salinity	Plants display heightened foliage coverage, larger fruit dimensions, improved root establishment, elevated nutrient concentrations, and increased antioxidant enzyme activity.	[113]
*Eucalyptus camaldulensis*	*Glomus* spp., *Gigaspora albida*, *Gigaspora decipiens*	Salinity	An increase in photosynthetic pigments, reduced leaf proline, and favorable effects on physiological and biochemical parameters were noted.	[114]
*Zea mays* L.	*Rhizophagus intraradices*, *Funneliformis mosseae*, *Funneliformis geosporum*	High temperature	Positive plant attributes, augmented photosynthetic transpiration rate, and improved pigments enhance overall growth.	[115]
*Triticum aestivum*	*Rhizophagus irregularis*, *Funneliformis mosseae*, *Funneliformis geosporum*, *Claroideoglomus claroideum*	High temperature	Improved nutrient uptake and increased grain numbers are among the observed effects.	[116]
*Zea mays* L.	*Glomus tortuosum*	Temperature stress	Elevated levels of shoot nitrogen (N), phosphorus (P), potassium (K), and copper (Cu), along with increased nitrate reductase activity, were recorded.	[117]
*Zea mays* L.	*Glomus tortuosum*	Cold stress	A notable increase in amino acid concentrations was observed.	[118,119]
*Elymus nutans* Griseb.	*Funneliformis mosseae*	Cold stress	Enhanced antioxidant enzymes, photosynthetic pigments, and overall plant growth were evident.	[120]
*Hordeum vulgare* L.	*Glomus versiforme*, *Rhizophagus irregularis*	Cold stress	The presence of elevated antioxidants, osmoprotectants, and increased potassium uptake positively impacted plant growth and metabolism of phenolics.	[121]
*Cucumis sativus* L.	*Rhizophagus irregularis*	Cold stress	Photosynthetic efficiency and carbon sink both showed improvement.	[122]
*Grapevine* (*Vitis vinifera* L.)	*Rhizoglomus irregulare*, *Funneliformis mosseae*	High-temperature stress	The growth rate increased alongside enhanced substrate carbon conversion efficiency and stomatal conductance.	[123]
*Zea maize* L.	*Funneliformis*	High temperature	Regulation of photosystem (PS) II heterogeneity was observed.	[115]
*Solanum lycopersicum*, *Capiscum annuum*, *Cucumis sativus*	*Rhizophagus irregularis*	High-temperature stress	Increased vigour, productivity, and fruit quality were prominent outcomes.	[124]
*Saccharum arundinaceum*	*Glomus* spp.	Drought	Enhanced levels of antioxidant enzymes, phenolics, chlorophyll, and plant biomass were found in leaves.	[125]
*Triticum aestivum*	*Glomus mosseae*	Drought	Improved chlorophyll levels, higher content of antioxidant enzymes ascorbic acid, and increased nitrogen (N), phosphorus (P), and potassium (K) content were noted.	[126]
*Ipomoea batatas*	*Glomus* spp.	Drought	Osmoprotectants played a role in adjusting osmotic potential.	[127]
*Lycopersicon esculatum*, *Capsicum annuum*	*Rhizophagus irregularis*, *Rhizophagus fasciculatus*	Drought	Increases in biomass, root and shoot length, and photosynthetic pigments were observed, while proline concentration decreased.	[128]
*Solanum lycopersicum*	*Funneliformis mosseae*, *Rhizophagus irregularis*	Drought	Enhanced plant height, stomatal conductance, water use efficiency, biomass, and reduced levels of reactive oxygen species (ROS) and abscisic acid (ABA) were recorded.	[129]
*Triticum aestivum* L.	*Glomus mosseae*, *Glomus fasciculatum*, *Gigaspora decipiens*	Drought	Positive effects on plant growth parameters and photosynthetic pigments were evident.	[130]
*Digitaria eriantha*	*Rhizophagus irregularis*	Drought	Increased shoot dry weight, stomatal conductance, lipid peroxidation, and ROS levels were observed in both shoot and root.	[131]
*Triticum durum*	*Rhizophagus intraradices*	Drought	Enhanced grain biomass, micronutrient content, and gliadins in grains were notable outcomes.	[132]
*Poncirus trifoliate*	*Funneliformis mosseae*, *Paraglomus occultum*	Drought	Growth attributes saw improvement through increased root weight and length, higher fructose and glucose levels, lower sucrose levels, and proline accumulation.	[133]
*Cupressus arizonica*	*Rhizophagus irregularis*, *Funneliformis mosseae*	Drought	Growth was enhanced, and levels of hydrogen peroxide and malondialdehyde were reduced.	[134]
*Ephedra foliata* Boiss	*Glomus etunicatum*, *Rhizophagus intraradices*, *Funneliformis mosseae*	Drought	Antioxidant enzyme activity, proline, glucose, and total soluble protein levels increased, improving nitrogen metabolism.	[135]
*Zea mays* L.	*Rhizophagus irregularis*	Drought	AM plant roots demonstrated diamine oxidase activity, converting putrescine into aminobutyric acid (GABA).	[136]
*Ceratonia silique*	*Glomus*, *Gigaspora*, *Acaulospora*, *Entrophospora*	Drought	Positive impacts included increased plant growth, nutrient levels, stomatal conductance, photosystem II (PSII) efficiency, and water content.	[137]
*Catalpa bungee* C.A.Mey	*Rhizophagus intraradices*	Drought	Root morphology, water content, biomass, photosynthetic pigments, and various plant hormones (except ABA) all showed improvement.	[138]
*Cinnamomum migao*	*Glomus lamellosum*, *Glomus etunicatum*	Drought	Enhanced antioxidant enzyme activity and osmoprotectants led to reduced malondialdehyde levels.	[139]
*Sesamum indicum* L.	*Funneliformis mosseae*, *Rhizophagus intraradices*	Drought	Improved oil and seed yield, total soluble protein, leaf phosphorus content, and heightened photosynthetic pigments were observed.	[140]
*Lonicera japonica* Thunb.	*Rhizophagus intraradices*, *Glomus versiforme*	Cd	Lower cadmium (Cd) levels in shoots and roots were noted, with more significant accumulation in roots. This indicated enhanced Cd tolerance.	[141]
*Solanum lycopersicum* L.	*Funneliformis mosseae*, *Rhizophagus intraradices*, *Claroideoglomus etunicatum*	Cd	Reduced malondialdehyde and ROS levels provided improved protection against Cd stress.	[142]
*Cajanus cajan* L.	*Rhizophagus irregularis*	Metals—cadmium and zinc	Increases in root biomass, macro- and micronutrients, and proline formation were observed.	[108]
*Zea mays* L.	*Glomus intraradices*	Heavy metal: cadmium	Combined effects were seen regarding soil alkalinization, Cd immobilization, and reduced Cd phytoavailability.	[143]
*Trigonella foenumgraecum*	*Glomus monosporum*, *Glomus clarum*, *Gigaspora nigra*	Metals—cadmium	Enhanced antioxidant enzyme activities and malondialdehyde content contributed to phytostabilization.	[144]
*Trigonella foenumgraecum*	*Glomus monosporum*, *Glomus clarum*, *Gigaspora nigra*	Cd	HX3 and HN89 plants showed no significant impacts on Cd accumulation or translocation.	[145]
*Glycine max*	*Rhizophagus irregularis*	Cd	Increased glomalin production and metal uptake were evident in plants.	[145]
*Helianthus annuus*	*Glomus mosseae*, *Glomus intraradices*	Cr, Mn, Ni, Cu, Zn, Al, Pb, Co, Mo, Fe, and Si	Root colonization was enhanced, increasing root and shoot dry weight and higher phosphorus content.	[146]
*Zea mays* L.	*Rhizophagus fasciculatus*, *Funneliformis mosseae*, *Rhizophagus intraradices*, *Glomus aggregatum*	Cd, Cr, Ni, Pb	Elevated levels of total chlorophyll content and net photosynthesis rate were observed.	[147]
*Phragmites australis*	*Funneliformis mosseae*	TiO_2_NPs	The plant’s biomass, growth, and physiological properties all experienced increases.	[148]
*Cynodon dactylon*	*Funneliformis mosseae*, *Diversisporas purcum*	Pb, Zn, Cd	The vulnerability of standing milkvetch to powdery mildew was enhanced, accompanied by improved shoot and root growth.	[149]
*Medicago sativa*	*Glomus aggregatum*, *Glomus intraradices*, *Glomus elunicatum*, *Glomus versiforme*	Cd	Dry weight, growth, yield, and production of antimicrobial substances all increased in plants.	[150]
*Medicago truncatula*	*Rhizophagus irregularis*	Pb	Reduced crop plant infections resulted in improved growth and yield.	[151]
*Phragmites australis*	*Rhizophagus irregularis*	Cu	Plant growth was heightened, along with an increase in functional leaf quantity.	[152]
*Sorghum vulgare*	*Acaulospora fragilissima*, *Acaulospora saccata*, *Claroideoglomus etunicatum*, *Pervetustus simplex*, *Rhizophagus neocaledonicus*, *Scutellospora ovalis*, *Rhizophagus neocaledonicus*	Ultramafic soils (Fe, Mn, Ni, Cr, and Co)	Growth hormone promotes plant health, increasing root and shoot weight and improving leaf structure.	[153]
*Solanum lycopersicum* L.	*Funneliformis mosseae*	*Cladosporium fulvum*	Positive outcomes include elevated phosphorus and nitrogen levels, enhanced seedling weight, and increased plant water content.	[154]
*Saccharum offcinarum* L.	*Gigaspora margarita*, *G. etunicatum*, *Scutellospora fulgida*	-	Enhanced growth, higher antioxidant enzyme activity, elevated proline and phenolic content, and improved uptake of vital mineral elements have been observed. Furthermore, the absorption of sodium ions was reduced.	[155]
*Astragalus adsurgens var. Shanxi Yulin*	*Claroideoglomus etunicatum*, *Glomus versiforme*, *Funneliformis mosseae*	*Erysiphe pisi* DC 1805	Among the crucial nutrients for plants, phosphorus (P), nitrogen (N), and potassium (K) play pivotal roles.	[156]
*Lycopersicon esculentum*, *Capsicum annuum*	*Rhizophagus irregularis*, *Rhizophagus fasciculatus*	*Fusarium oxysporum* f. sp. *lycopersici*	Benefits encompass improved plant and root systems, heightened nutrient uptake, minimized lipid peroxidation, and reduced membrane damage.	[157]
*Capsicum annum*	*Glomus* spp.	*Pythium aphanidermatum*	Plant growth sees improvements through elevated antioxidant enzymes, increased photosynthetic compounds, soluble sugars, and proline content.	[158]
*Glycine max* (L.) Merr	*Rhizophagus irregularis*	*Macrophomina phaseolina*	Enhanced biomass, chlorophyll content, nutrient absorption, and accumulation of compatible osmolytes contribute to overall plant well-being.	[159]

## Data Availability

Not applicable.
